# Supplementary material for: Polyketide synthase gene clusters in the ericoid mycorrhizal fungus Oidiodendron maius: genome-wide prediction, expression profiling, and functional characterization of knockout mutants
Source: Front Plant Sci. 2026 Sep 16;17:1924430. doi: 10.3389/fpls.2026.1924430 (PMC13623903; doi:10.3389/fpls.2026.1924430)
Supplement: Supplementary file 1 [file SupplementaryFile1.zip › Supplementary Materials/Supplementary Folder 1.zip!/Oidma1_AssemblyScaffolds_Repeatmasked/knownclusterblast/cluster40/jgi.p_Oidma1_148698_mibig_hits.html]

| MIBiG Protein | Description | MIBiG Cluster | MiBiG Product | % ID | % Coverage | BLAST Score | E-value |
| --- | --- | --- | --- | --- | --- | --- | --- |
| AIE77069.1 | methyltransferase | BGC0000418 | Ristocetin\_biosynthetic\_gene\_cluster | 27 | 91.9860627178 | 86 | 5.8e-17 |
| AIG79231.1 | Hypothetical\_protein | BGC0000419 | Ristomycin\_A\_biosynthetic\_gene\_cluster | 27 | 94.0766550523 | 85 | 1.3e-16 |
| CBZ42134.1 |  | BGC0001117 | Himastatin\_biosynthetic\_gene\_cluster | 37 | 52.2648083624 | 82 | 1.1e-15 |
